# The novelty of research article: Whose responsibility is it?

**DOI:** 10.4103/0253-7613.75689

**Published:** 2011-02

**Authors:** Ravindra S. Beedimani

**Affiliations:** American University of Caribbean, School of Medicine, St. Maarten, Netherlands Antilles E-mail: ravisb123@gmail.com

Sir,

I would like to comment on the article entitled “Effective control of sickle cell disease with hydroxyurea therapy”[1] published in Jan-Feb, 2010 issue of *Indian Journal of Pharmacology*. I have found nothing novel about the topic which makes it to be published. Any research article must meet certain criteria and standards that are set by the editorial and review committee before it can be published. That said, I saw nothing novel in the study, the main objective of which was the evaluation of hydroxyurea therapy in sickle cell patients. This has already been well studied and established for some time;[2,3] indeed, this is referenced in the article itself and in standard pharmacology texts.[4,5] In the 15 years that I have instructed undergraduate and postgraduate medical students in Pharmacology, I have routinely taught about the effectiveness and safety of hydroxyurea in the control of sickle cell disease. I would therefore enquire the authors and other concerned members about the criteria that merited publication of the article in the journal.

## References

[1] Singh H, Dulhani N, Kumar BN, Singh P, Tiwari P (2010). Effective control of sickle cell disease with hydroxyurea therapy. Indian J Pharmacol.

[2] Ware RE, Zimmerman SA, Schultz WH (1999). Hydroxyurea as an alternative to blood transfusions for the prevention of recurrent stroke in children with sickle cell disease. Blood.

[3] Platt OS, Orkin SH, Dover G, Beardsley GP, Miller B, Nathan DG (1984). Hydroxyurea enhances fetal hemoglobin production in sickle cell anemia. J Clin Invest.

[4] Cokic VP, Smith RD, Beleslin-Cokic BB, Njoroge JM, Miller JL, Gladwin MT (2003). Hydroxyurea induces fetal hemoglobin by the nitric oxide-dependent activation of soluble guanylyl cyclase. J Clin Invest.

[5] Franco RS, Yasin Z, Palascak MB, Ciraolo P, Joiner CH, Rucknagel DL (2006). The effect of fetal hemoglobin on the survival characteristics of sickle cells. Blood.

